# Symptoms of Laceration of the Womb

**Published:** 1856-08

**Authors:** Edwin R. Maxson

**Affiliations:** Geneva, N. Y.


ART. VI.—*Symptoms of Laceration of the Womb.* By EDWIN R. MAXSON, M. D., of Geneva, N. Y.

As rupture of the womb is liable to occur during labor, it becomes a matter of importance to be able, by the symptoms, to ascertain the fact before the child escapes into the peritoneal sac.

The womb may be lacerated at almost any point; but the inferior half of the left side is the point at which, according to my observation, it most frequently occurs, the rupture sometimes extending through the cervix, and into the vagina, but in other cases being confined to the body of the womb.

I know of no reason why this should be the most frequent seat of laceration, unless it be from the fact that the patient more frequently lies on the left side during labor, thus bringing greater tension upon that part.

If the extent of the laceration be considerable, the child may escape at once into the peritoneal sac; but if, as is sometimes the case, the extent of the rupture be not great at first, the child may be retained in the cavity of the uterus for some time at least. Hence the importance of understanding the symptoms of laceration at once, even though it be of limited extent, so that, if possible, delivery may be effected before the child is expelled from the womb into the peritoneal sac.

If a laceration of the womb occur, of considerable extent, there is a severe pain felt in the part, distinct from the labor pain: though it occurs during a severe labor pain. There is a sudden flow of blood, of greater or less extent, according to the seat and extent of the rupture.

The labor pains generally cease at once, or very soon, and the child, if it has escaped from the womb, may be felt in its new position by palpation over the abdomen, or by an examination per vaginam.

The hæmorrhage though quite profuse at first, may subside in twenty or thirty minutes; and the severe tearing pain gradually becomes a dull, heavy pain, accompanied with a feeling of oppression, the patient appearing to suffer great distress from any considerable motion of the body.

By attending to these symptoms we may save ourselves the mortification, and our patients the inconvenience of resorting to measures or remedies to revive the sinking labor pains, or to allay the severe pain which the laceration has produced, and thus, also, avail ourselves of the earliest possible time for extricating both mother and child from their unfortunate condition.

If, however, a laceration of the womb occur, not of an extent sufficient to admit of the expulsion of the child through the rupture into the peritoneal sac, an early knowledge of the fact is of equal or greater importance; and it may generally be ascertained by careful attention to the symptoms which are developed.

There is a sudden flow of blood which may, however, soon become less and gradually subside. The patient complains of oppression, with more or less faintness; and if an examinatian be made, the child will usually be found above the superior strait, and without any apparent disposition to engage.

Such were the symptoms in a case of this character to which I was called a few months since, by my friend Dr. H. A. Potter, of this village: he suspecting a laceration of the womb, immediately on the occurrence of the symptoms which I have here laid down.

Now, by careful observance of the symptoms, immediately on their development, I am confident that the real condition may generally be ascertained while yet the laceration is not of an extent sufficient to allow the passage of the child into the peritoneal sac. And by adopting a judicious course of procedure thus early, some cases at least may be conducted to a favorable issue.

If it be a head presentation, and the head can be reached and the child delivered by the forceps, the case may terminate favorably, if too much blood has not escaped into the peritoneal sac.

If it be a breech presentation, the feet may be sought, and thus a safe delivery effected.

If, however, the laceration be extensive and the child has escaped into the peritoneal sac, and cannot readily be reached, I believe that delivery by a Cæsarian operation offers the best chance of a favorable issue of the case.

GENEVA, N. Y., May 27, 1856.

